# Long-Term Increased Carnitine Palmitoyltransferase 1A Expression in Ventromedial Hypotalamus Causes Hyperphagia and Alters the Hypothalamic Lipidomic Profile

**DOI:** 10.1371/journal.pone.0097195

**Published:** 2014-05-12

**Authors:** Paula Mera, Joan Francesc Mir, Gemma Fabriàs, Josefina Casas, Ana S. H. Costa, Maria Ida Malandrino, José-Antonio Fernández-López, Xavier Remesar, Su Gao, Shigeru Chohnan, Maria Sol Rodríguez-Peña, Harald Petry, Guillermina Asins, Fausto G. Hegardt, Laura Herrero, Dolors Serra

**Affiliations:** 1 Department of Biochemistry and Molecular Biology, Facultat de Farmàcia, Universitat de Barcelona and Institut de Biomedicina de la Universitat de Barcelona (IBUB) and CIBER Fisiopatología de la Obesidad y la Nutrición (CIBERobn), Instituto de Salud Carlos III, Barcelona, Spain; 2 Research Unit on BioActive Molecules, Department of Biomedicinal Chemistry, Institute of Advanced Chemistry of Catalonia (IQAC)/CSIC, Barcelona, Spain; 3 Department of Nutrition and Food Science, Facultat de Biologia, Universitat de Barcelona and Institut de Biomedicina de la Universitat de Barcelona (IBUB) and CIBER Fisiopatología de la Obesidad y la Nutrición (CIBERobn), Instituto de Salud Carlos III, Barcelona, Spain; 4 Scripps Research Institute, Jupiter, Florida, United States of America; 5 Department of Bioresource Science, College of Agriculture, Ibaraki University, Ibaraki, Japan; 6 UniQure, Amsterdam, The Netherlands; Institut d'Investigacions Biomèdiques August Pi i Sunyer, Spain

## Abstract

Lipid metabolism in the ventromedial hypothalamus (VMH) has emerged as a crucial pathway in the regulation of feeding and energy homeostasis. Carnitine palmitoyltransferase (CPT) 1A is the rate-limiting enzyme in mitochondrial fatty acid β-oxidation and it has been proposed as a crucial mediator of fasting and ghrelin orexigenic signalling. However, the relationship between changes in CPT1A activity and the intracellular downstream effectors in the VMH that contribute to appetite modulation is not fully understood. To this end, we examined the effect of long-term expression of a permanently activated CPT1A isoform by using an adeno-associated viral vector injected into the VMH of rats. Peripherally, this procedure provoked hyperghrelinemia and hyperphagia, which led to overweight, hyperglycemia and insulin resistance. In the mediobasal hypothalamus (MBH), long-term CPT1AM expression in the VMH did not modify acyl-CoA or malonyl-CoA levels. However, it altered the MBH lipidomic profile since ceramides and sphingolipids increased and phospholipids decreased. Furthermore, we detected increased vesicular γ-aminobutyric acid transporter (VGAT) and reduced vesicular glutamate transporter 2 (VGLUT2) expressions, both transporters involved in this orexigenic signal. Taken together, these observations indicate that CPT1A contributes to the regulation of feeding by modulating the expression of neurotransmitter transporters and lipid components that influence the orexigenic pathways in VMH.

## Introduction

Current lifestyles are responsible for the alarming increase in the prevalence of obesity and the consequent development of insulin resistance and Type 2 diabetes. An imbalance between energy intake and expenditure can cause overweight, thus contributing to obesity and associated metabolic complications. The hypothalamus is crucial to the central control of appetite and energy homeostasis [1], [2]. This brain region consists of interconnected neuronal nuclei that respond to neuroendocrine and metabolic signals by modulating the production and release of specific neurotransmitters that control energy balance [3]. Hypothalamic lipid metabolism participates in this process and is linked to the molecular mechanisms by which hormones, such as leptin, ghrelin and insulin, exert their central effect on food intake [4]–[6].

Malonyl-CoA, the first intermediate in fatty acid (FA) biosynthesis, has emerged as a crucial player in the hypothalamic control of feeding [7], [8]. On the one hand, decreased food intake and increased malonyl-CoA are observed after central treatment of drugs or anorectic hormones such as leptin. Leptin's anorectic pathway involves the inhibition of AMP-activated protein kinase (AMPK), which, in turn, activates acetyl-CoA carboxylase (ACC), key enzyme for malonyl-CoA synthesis [5]. Treatments with FAS inhibitors, such as C75 and cerulenin reduce food intake by an increase of hypothalamic malonyl-CoA level [8]–[10]. On the other hand, malonyl-CoA level decreases under fasting condition when ghrelin level is high. Orexigenic ghrelin pathway involves activation of AMPK, inhibition of ACC and a reduction of malonyl-CoA level [11], [12]. One clear candidate for malonyl-CoA action is carnitine palmitoyltransferase (CPT) 1, a key enzyme regulating mitochondrial long chain fatty acyl-CoA (LCFA-CoA) β-oxidation [13], since CPT1 activity is physiologically inhibited by malonyl-CoA. An accumulation of LCFA-CoA in the hypothalamus was believed to signal reduction in food intake and hepatic gluconeogenesis in rodents. CPT1 is related to both metabolites and it has been implicated in the central control of both food intake and glucose metabolism [14]–[17]. Two CPT1 isoforms are expressed in the hypothalamus, CPT1A and CPT1C. The latter is found mainly in the endoplasmic reticulum of neurons and does not directly participate in mitochondrial FA β-oxidation (FAO) [18]. However, CPT1C binds malonyl-CoA and it may serve as a sensor for malonyl-CoA in the hypothalamic regulation of energy homeostasis [19], [20]. Furthermore, we have recently shown that CPT1C mediates ghrelin central action by altering ceramide levels [21]. CPT1A also contributes to the central orexigenic action of ghrelin, since the molecular events derived from ghrelin binding to its receptor on hypothalamic neurons result in increased CPT1A activity and FAO [6], [22], [23]. It has been proposed that the derived metabolic changes, including the accumulation of reactive oxygen species (ROS) and the subsequent up-regulation of the mitochondrial uncoupling protein 2 (UCP2), contribute to the activation of arcuate (Arc) AgRP neurons [22]. Moreover, transcription factors such as brain-specific homeobox (Bsx), cAMP response-element binding protein (CREB), and forkhead box O1 (FoxO1) act as downstream mediators of CPT1A in the Arc nucleus for orexigenic neuropeptide synthesis [24]. These observations suggest a potential role of hypothalamic CPT1A in the control of feeding, however the exact mechanistic sequence and mediators involved are not yet revealed.

A growing body of evidence implicates the ventromedial hypothalamus (VMH) in the central control of food intake and regulation of energy homeostasis [17], [25], [26]. VMH neurons are reported to activate anorexigenic neuronal pathways in the Arc nucleus by projecting excitatory inputs into POMC neurons [27]. Moreover, some VMH neurons are GABAergic [28]. We have recently observed that CPT1A activity in the VMH changes concomitantly with fasting and refeeding states and that it is reinforced by the increase in appetite provoked by acute expression of a permanently activated CPT1A isoform [29]. Despite all the evidence, the exact mechanisms for the induction of feeding downstream of CPT1A in the VMH are unknown.

Here we examined the long-term effect of AAV-vectorized expression of a malonyl-CoA-insensitive CPT1A isoform [30], namely CPT1AM, in the VMH. This model allows us to uncouple malonyl-CoA effect on food intake and to activate permanently downstream effectors of CPT1A involved in feeding. We hypothesise that CPT1A modulates the lipidic and gene profile of the mediobasal hypothalamus (MBH, encompassing both Arc and the VMH), which may be involved in the central control of feeding and glucose metabolism. Long-term CPT1AM expression led to alterations in MBH structural bioactive lipids, i.e. phospholipids, sphingolipids and ceramides. In addition, this CPT1AM expression altered the expression of glutamate and GABA vesicular transporters, which have been reported to control amino acid neurotransmitters which alter food intake. Moreover, hyperphagia, overweight and the later development of insulin resistance and hyperglycemia was also observed in the CPT1AM animals. All these results reinforce the notion that VMH CPT1A is involved in appetite modulation.

## Materials and Methods

### Adeno-associated vectors (AAVs)

Serotype 1-AAV, AAV-GFP and AAV-CPT1AM were constructed to express GFP and CPT1AM respectively. Vector plasmids carried: CMV promoter, cDNA sequence of GFP or CPT1AM [30], woodchuck posttranscriptional regulatory element (WPRE, acc #AY468–486) [31], and bovine growth hormone polyadenosine transcription termination signal (bases 2326–2533 GenBank acc #M57764). The expression cassette was flanked by two inverted terminal repeats (ITRs) derived from serotype 2-AAV. AAVs were produced in insect cells using a baculovirus [32]. The vector preparation used had the following titers: AAV-GFP, 5×10^12^ pfu/mL; and AAV-CPT1AM, 2.5·10^12^ pfu/mL.

### Animals

The *Comité Ètic d’Experimentació Animal de la Universitat de Barcelona (CEEA-UB)* and the *Generalitat de Catalunya Department de Medi Ambient i Habitatge,* in accordance with current legislation, approved all experimental protocols from this work (Permit Numbers: 4068 and 5471). Sprague-Dawley male rats (260–290 g) (Harlan Co. Laboratories) were used in all the studies. Animals were housed in individual cages and maintained under a 12 h dark/light cycle with free access to food (2014, Harlan) and water. Rats were anaesthetised with intraperitoneal ketamine (Imalgene, 90 mg/kg) and xylazine (Rompun, 11 mg/kg) and immobilised in a stereotactic apparatus. Chronic catheters (26-gauge stainless steel guide cannulae (Plastic one)) were implanted bilaterally in the VMH (coordinates from Bregma: −2.8 mm posterior, ±0.7 mm lateral and −10 mm ventral [33]). During the week after the surgery, animals received analgesics (buprenorphine, 0.3 mg/400 mL) and antibiotics (enrofloxacin, 10%) with water to aid recovery. Next, rats with VMH cannulae were given bilateral injections (1 µL/each site) of AAV-GFP (control) or AAV-CPT1AM at a rate of 0.2 µL/min. Food intake and body weight were measured in rats infected with AAV-CPT1AM (hereafter CPT1AM animals) and AAV-GFP (hereafter GFP animals) in the VMH.

### Glucose Tolerance Test (GTT)

The GTT was performed in conscious rats 14 weeks after the AAV injection in the VMH. Glucose (2.0 g per kg body weight) was administered intraperitoneally after an overnight fast (16 h), and blood glucose concentrations were measured using a Glucometer Elite (Bayer) at baseline and 15, 30, 60, 90 and 120 min after glucose administration.

### Measurement of circulating hormones and metabolites

Blood was collected from rats and processed to provide plasma and serum. Commercial kits were used to measure serum insulin (Rat/Mouse Insulin ELISA (Millipore)), leptin (Mouse/Rat Leptin ELISA (B-Bridge)), adiponectin (Rat Adiponectin ELISA (Millipore)), non-esterified FA (NEFA) (Wako Chemicals), plasma acylated-ghrelin (Rat Acylated-Ghrelin ELISA (BioVendor)), T3, T4, and TSH (ELISA (DRG Diagnostics)). For the measurement of plasma amino acids, distilled water (100 µL), 1000 µM NLE (50 µL) and (trifluoracetic acid) 10%TFA acid (100 µL) were added to 100 µL plasma sample. After a 10-min incubation, tubes were centrifuged at 10000×*g*. The supernatant was filtered (Ultracel membrane 10 KDa filter (Millipore)), dried under a N_2_ stream, and redissolved in lithium citrate pH 2.2 (400 µL). Amino acids were measured at the Scientific-Technical Services of the University of Barcelona using an auto-analyser (Biochrom 30).

### Determination of liver triacylglyceride (TAG) content

Pulverised frozen tissue from rats (≈100 mg) was homogenised in 500 µL PBS. Lipids were extracted using chloroform, dried under a N_2_ stream, and redissolved in n-propanol. TAGs were quantified using the Triglycerides Determination Kit (Sigma Aldrich).

### Determination of malonyl-CoA, LCFA-CoA and acylcarnitine content

MBH wedges (encompassing Arc and VMH nuclei) and liver were quickly removed, frozen in liquid nitrogen, and stored at −80 °C prior to malonyl-CoA or LCFA-CoA quantification. The former was measured using a malonyl-CoA recycling assay as described elsewhere [8], [34]. LCFA-CoAs were extracted and measured by HPLC-MS/MS at the Scientific-Technical Services of the University of Barcelona, as previously described [35]. Acylcarnitines were analysed using an Acquity UPLC-TOF system (Waters) with an BEH C8 column (1.7 µm particle size, 100 mm×2.1 mm, Waters). The two mobile phases were 1 mM ammonium formate in methanol (phase A) and 2 mM ammonium formate in H2O (phase B), both phases with 0.05 mM formic acid. The following gradient was programmed: 0 min, 65% A; 10 min, 90% A; 15 min, 99% A; 17 min, 99% A; 20 min, 65% A, and a flow rate of 0.3 mL min-1. Quantification was carried out using the extracted ion chromatogram of each compound, using 50-mDa windows. The linear dynamic range was determined by injecting standard mixtures. Positive identification of compounds was based on the accurate mass measurement with an error <5 ppm and their LC retention time compared to that of a standard (±2%).

### Lipidomic analysis

MBH wedges were quickly removed, frozen in liquid nitrogen, and stored at −80 °C prior to lipid analysis. Sphingolipid extraction and analysis by UPLC-TOF was carried out as described [36]. Phospholipid extracts were obtained using the same procedure but without the saponification step. Lipids were analysed by UPLC-TOF in positive or negative mode. The two mobile phases were 1 mM ammonium formate in methanol (phase A) and 2 mM ammonium formate in H_2_O (phase B), both phases with 0.05 mM formic acid. The following gradient was programmed: 0 min, 80% A; 3 min, 90% A; 6 min, 90% A; 15 min, 99% A; 18 min, 99% A; 20 min, 80% A, and a flow rate of 0.3 mL min^−1^.

### Histological analysis

Brain histological examination was done using 50- μm thick sections. Brains were excised and fixed overnight in 10% neutral-buffered formalin (Sigma). Next, they were immersed in 20% sucrose phosphate-buffered solution (PBS; pH 7.0) for 18–36 h at 4 °C. Coronal sections were obtained using a freezing-sliding microtome and mounted onto microscope slides using Immu-Mount (Thermo) to prevent fading. Examination of white adipose tissue (WAT), brown adipose tissue (BAT), and liver histology were done using 4- μm thick formalin-fixed, paraffin-embedded tissue sections stained with hematoxylin and eosin (H&E) at the Pathology Department of *Hospital Clínic* of Barcelona.

### mRNA expression analysis

The MBH, liver, WAT, and BAT from GFP and CPT1AM rats were excised, frozen, and stored at -80 °C. Total RNA was isolated from frozen MBH, WAT, and BAT using RNeasy Lipid Tissue Mini-Kit (Qiagen) and from frozen liver using RNeasy Mini-Kit (Qiagen). cDNA was synthesised using the Transcriptor First Strand cDNA Synthesis Kit (Roche). *q*RT-PCR analyses were performed in a LightCycler 480 Instrument (Roche). To discern between the endogenous CPT1A (*CPT1Awt*) and the expressed isoform CPT1AM, we used specific primers and FRET probes and a LightCycler 480 Probes Master (Roche). The mRNA expression of other genes was determined using intron-skipping primers and SYBR Green Master Mix (Applied Biosystems). All sequences are available upon request.

### Statistical analysis

Data are expressed as mean ± SEM. Statistical significance was determined by ANOVA and Student's *t* test, using Microsoft Excel and GraphPad Prism 6 software. A p value < 0.05 was considered significant.

## Results

### AAV-mediated expression of GFP and CPT1AM

**Figure 1:**
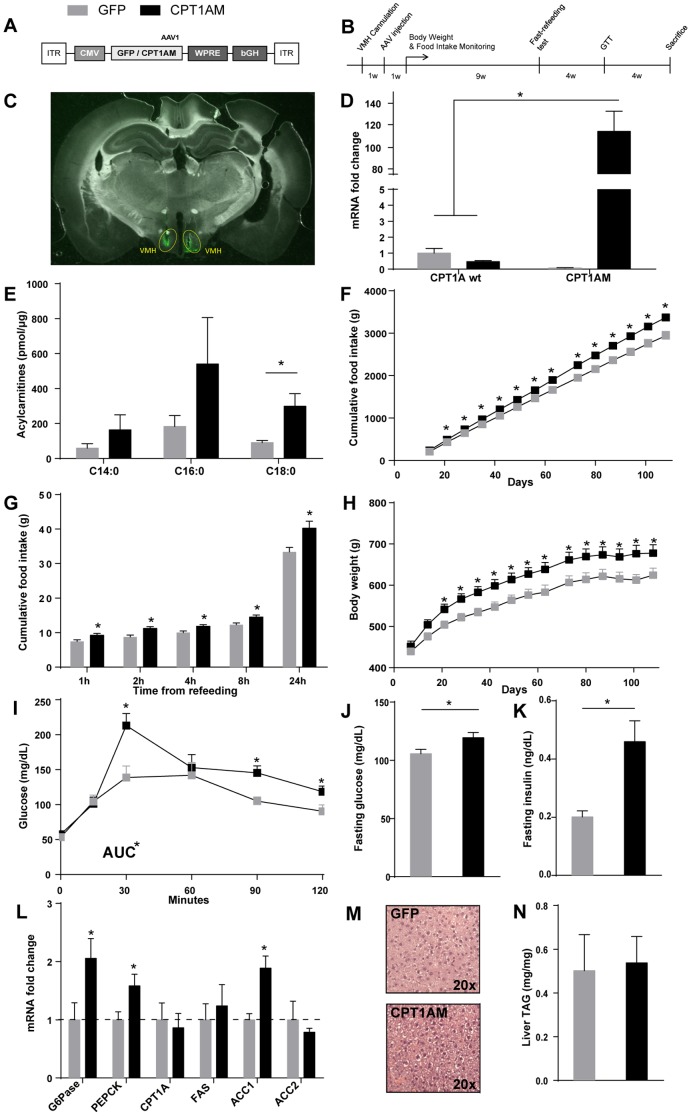
Analysis of the effect of VMH CPT1A expression on feefing behaviour and glucose homeostasis. (A) Scheme of the AAV vectors used in this study: AAV-GFP and AAV-CPT1AM. The cassettes contain the GFP or CPT1AM transgene driven by the cytomegalovirus (CMV) promoter. (B) Scheme of the time course of the experiment. (C) Representative histological section showing GFP in the VMH of GFP rats 2 weeks after the bilateral injection of AAV vector. (D) CPT1Awt and CPT1AM mRNA expression in the MBH (VMH + Arc) of GFP and CPT1AM rats. Measurements were performed 18 weeks after the bilateral injection of AAV vectors. Primers specifically recognise the sequence of wt or mutant CPT1A. (E) Acylcarnitines were measured as an indirect parameter of CPT1A activity. *n* = 3 (F) Cumulative food intake, (G) fast-refeeding test and (H) body weight change in rats fed a normal diet. *n* = 11. In E and G, X-axis represents days after the bilateral injection of AAV vectors into the VMH. (I) Intraperitoneal GTT in GFP and CPT1AM animals after an overnight fasting. The test was performed in animals 14 weeks after the AAV-injection into the VMH. (J) Blood fasting glucose and (K) serum fasting insulin levels measured in GFP and CPT1AM rats 18 weeks after the bilateral injection of AAV vectors into the VMH. The following analyses were performed 18 weeks after the AAV-injection into the VMH. (L) Liver relative mRNA expression of genes associated with gluconeogenesis and lipid metabolism in GFP and CPT1AM rats. (M) Liver histological sections (H & E staining) from representative GFP and CPT1AM rats. (N) Liver TAG content in GFP and CPT1AM animals. *n* = 5–6 animals in all cases. Error bars represent SEM. *p<0.05. AUC: area under the curve.

AAV carrying CPT1AM or GFP were obtained for long-term expression of the permanently active form of CPT1AM or GFP (Fig. 1A). AAV vectors were bilaterally injected into the VMH and several experiments were performed according to the showed scheme (Fig. 1B). Histological studies in GFP rats revealed that AAV-infected cells in the hypothalamus were limited mainly to the VMH (Fig. 1C). *q*RT-PCR analyses performed in MBH showed a robust 113±19.2-fold increase (*p*<0.0001) in the CPT1AM mRNA in CPT1AM rats with respect to GFP control rats (Fig. 1D). We analysed the levels of the long-chain acylcarnitines, as direct products of CPT1A activity in MBH samples. The levels of C18:0-acylcarnitine increased 3.3-fold in CPT1AM (298.4±72.62 pmol/μg) compared to GFP-expressing counterparts (90.46±12.69 pmol/μg, *p*<0.05). C14:0- and C16:0-acylcarnitine levels also increased, but not significantly (Fig. 1E).

### CPT1AM expression in the VMH increased food intake and led to obesity, hyperglycemia and insulin resistance

We monitored the food intake and body weight of CPT1AM and GFP rats fed regular chow. The former group showed hyperphagia compared to GFP animals. Cumulative food intake was significantly higher 20 days after AAV injection (912±30.7 *vs.* 798±17.8 g, *p*<0.05) This increased food intake was maintained until the sacrifice (Fig. 1F). Furthermore a fast-refeeding test was performed to discern if this increased food intake may be due to impaired satiety. CPT1AM rats showed increased food intake in all measures performed after refeeding (Fig. 1G). Body weight was also measured. CPT1AM rats showed a significantly higher (83.8±13.7 g *vs.* 57±30.7 g, *p*<0.05) body weight change 20 days after AAV injection (Fig. 1H).

Glucose tolerance, blood glucose and serum insulin concentrations were examined in fasted CPT1AM and GFP rats. Fourteen weeks after the AAV injection, the GTT demonstrated glucose intolerance in the former (Fig. 1I). When sacrificed, CPT1AM rats showed higher fasting glucose (increase of 16.4±3.3%, *p*<0.01) and insulin (increase of 31.6±9.2%, *p*<0.01) than GFP animals (Fig. 1J and 1K). Furthermore, the expression of key gluconeogenic enzymes, such as glucose-6-phosphatase (G-6-Pase) and phosphoenolpyruvate carboxykinase (PEPCK), was analysed in the liver of fasted GFP and CPT1AM animals (Fig. 1L). Results showed a 2±0.3-fold and 1.6±0.5-fold increase in G-6-Pase and PEPCK mRNA respectively in CPT1AM animals with respect to controls (*p*<0.05). These results correlate with the hyperglycemia observed in the CPT1AM rats (Fig. 1J). The analysis of liver mRNA levels of lipid metabolism-related genes revealed a 2.1±0.3-fold increase in acetyl-CoA carboxylase 1 (ACC1) in CPT1AM rats (*p*<0.01) (Fig. 1L). However, we found no significant changes in the hepatic concentration of TAG (Fig. 1N). In addition, histological liver examination revealed no major differences in the hepatic anatomy of between the two groups (Fig. 1M).

**Figure 2:**
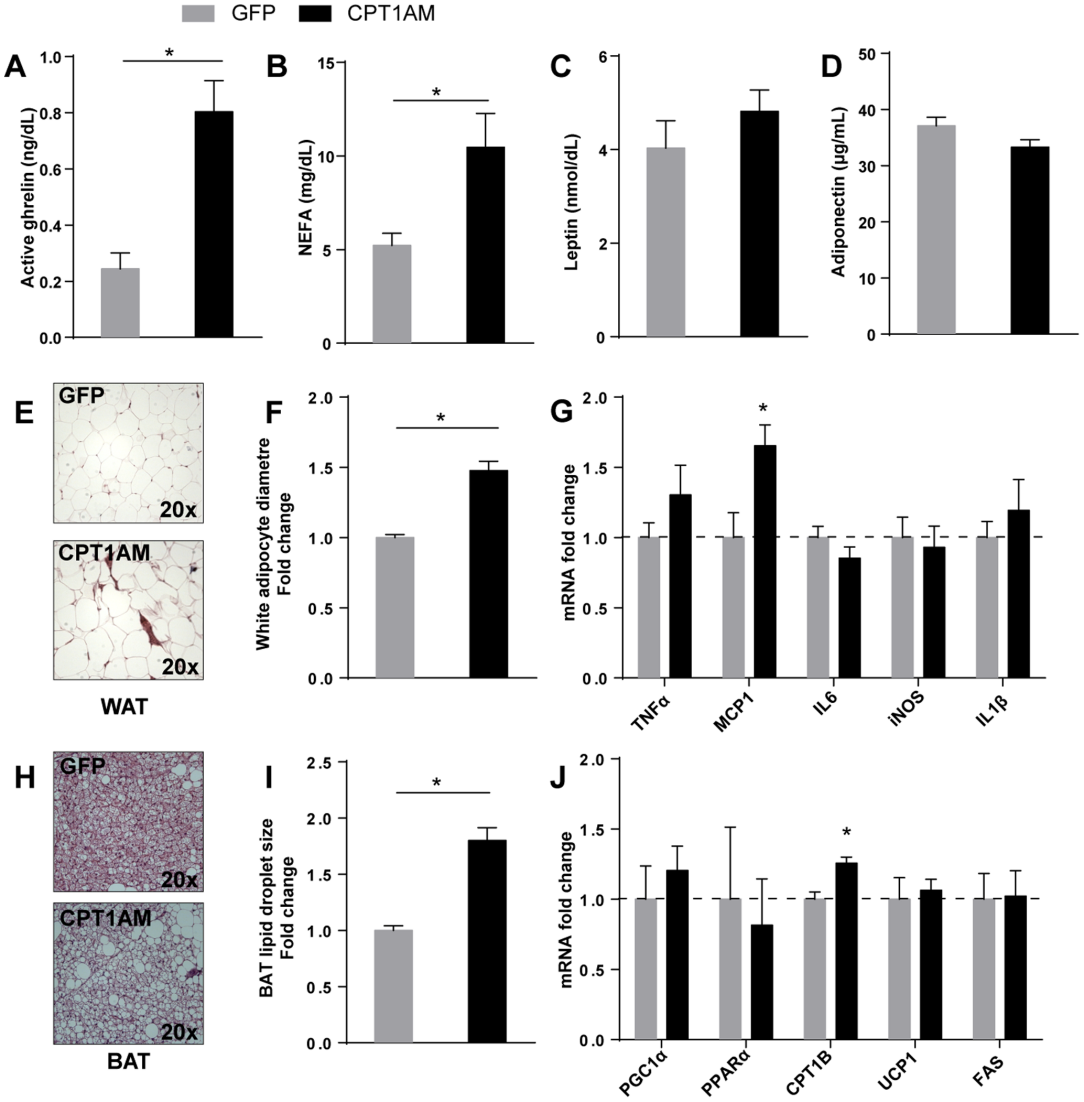
Analysis of postprandial metabolic and hormonal profile, adipose tissue histology, and mRNA expression in GFP and CPT1AM rats. Serum and plasma samples from fed GFP and CPT1AM rats were used for all the analyses. (A) Plasma active ghrelin. (B) Serum non-esterified fatty acids (NEFA). (C) Serum leptin. (D) Serum adiponectin. *n* = 6–10 animals per group. (E) Epididymal WAT histological sections (H & E staining) from representative GFP and CPT1AM rats. (F) White adipocyte diameter scored using ImageJ software and expressed as a fold change respect to GFP animals (5 high power field counted per rat, *n* = 3 rats per group). (G) mRNA expression of pro-inflammatory markers in WAT from GFP and CPT1AM rats. *n* = 5–7 animals per group. (H) Interscapular BAT histological sections (H & E staining) from representative GFP and CPT1AM rats. (I) Lipid droplet size scored using ImageJ software and expressed as a fold change respect to GFP animals (4 high power field counted per rat, *n* = 3 rats per group). (J) mRNA expression of genes associated with lipid metabolism and thermogenesis in BAT from GFP and CPT1AM animals. *n* = 10 animals per group. Error bars indicate SEM. *p<0.05.

Next we studied the circulating metabolic and hormonal profile of GFP and CPT1AM rats. In parallel with the increase in food intake, fed CPT1AM animals presented a significant rise (3.3±1.4-fold increase, *p*<0.001) in post-absorptive plasma levels of ghrelin (octanoylated/active form) (Fig. 2A). In addition, they exhibited high serum NEFA levels (2±0.3-fold increase, *p*<0.05) (Fig. 2B) equivalent to those of fasted GFP and CPT1AM counterparts (data not shown). Levels of other hormones associated with energy metabolism, such as thyroid hormones (T3, T4 and TSH, data not shown), leptin and adiponectin, were unaltered between the two groups (Fig. 2C, 2D). In addition, we found significant differences in the plasma aminogram of the two groups of animals (Table 1). Interestingly, specific branched-chain amino acid (BCAA), such as Val and Ile, considered markers of obesity, increased by 18.8% and 19.8% respectively in CPT1AM animals (Table 1). Obesity is characterised by hyperplasia in WAT and britening of BAT. Histological examination of epididymal WAT revealed that white adipocytes from CPT1AM animals were significantly larger than those of the GFP controls (Fig. 2E and F). Analyses of the mRNA expression of pro-inflammatory cytokines showed an up-regulation of the monocyte chemoattractant protein-1 (MCP1) (1.6±0.4-fold increase, *p*<0.05) in CPT1AM WAT with respect to GFP WAT (Fig. 2G). BAT showed a brown-to-white transformation in CPT1AM rats (Fig. 2H and I). We also determined the mRNA expression of genes associated with thermogenesis and FA metabolism in BAT. A moderate increase (1.2±0.1-fold, p<0.01) in the expression of CPT1B in CPT1AM rats was detected with respect to GFP controls (Fig. 2J). All these results indicate that the expression of CPT1AM in the VMH impairs satiety and leads to an obesogenic phenotype.

**Table 1:** Analysis of plasma amino acids from CPT1AM and GFP rats.

	GFP-expressing rats	CPT1AM-expressing rats	
**Amino acids** (μM)	*n* = 6	*n* = 6	*p* value
Taur	132±9	128±10.1	*ns*
Asp	23.7±0.8	26.7±1.2	*ns*
Hypro	1.6±0.2	1.7±0.1	*ns*
Thr	198.5±11.1	216.7±9.6	*ns*
Ser	216.2±6	221.4±5.6	*ns*
Asn	30.4±1.1	30.5±2.3	*ns*
Glu	192.4±15.8	265.1±12.3	*ns*
Gln	103.1±9.8	91.3±10.1	*ns*
Pro	70.8±1.5	67.5±2.7	*ns*
Gly	251±7.4	223.1±8.3	0.04
Ala	335±11.3	324.3±10.7	*ns*
Citr	66.4±3.5	71.2±3.8	*ns*
Val	159.4±6.2	189±5.8	0.006
Cyst	50±1.4	57.1±3.1	*ns*
Met	78.5±2.4	77.1±4.5	*ns*
Ile	66.1±2.5	79.2±2.2	0.003
Leu	194.3±6.9	204.9±3	*ns*
Tyr	127.5±8.4	144.7±8	*ns*
Phe	75±2.8	79.7±0.9	*ns*
Orn	115.5±3.2	163±7.1	0.004
Lys	632±9.1	696.1±25	0.05
His	101.3±2.7	122.4±2.8	0.001
Trp	21.9±1.5	20.8±2	*ns*
Arg	215.4±9.4	225.9±6.3	*ns*

Analysis was performed on plasma obtained at 18 weeks after AAV injection. Data indicated as Mean ± SEM.

### CPT1AM expression in the VMH alters MBH mRNA levels of different genes involved in food intake

**Figure 3:**
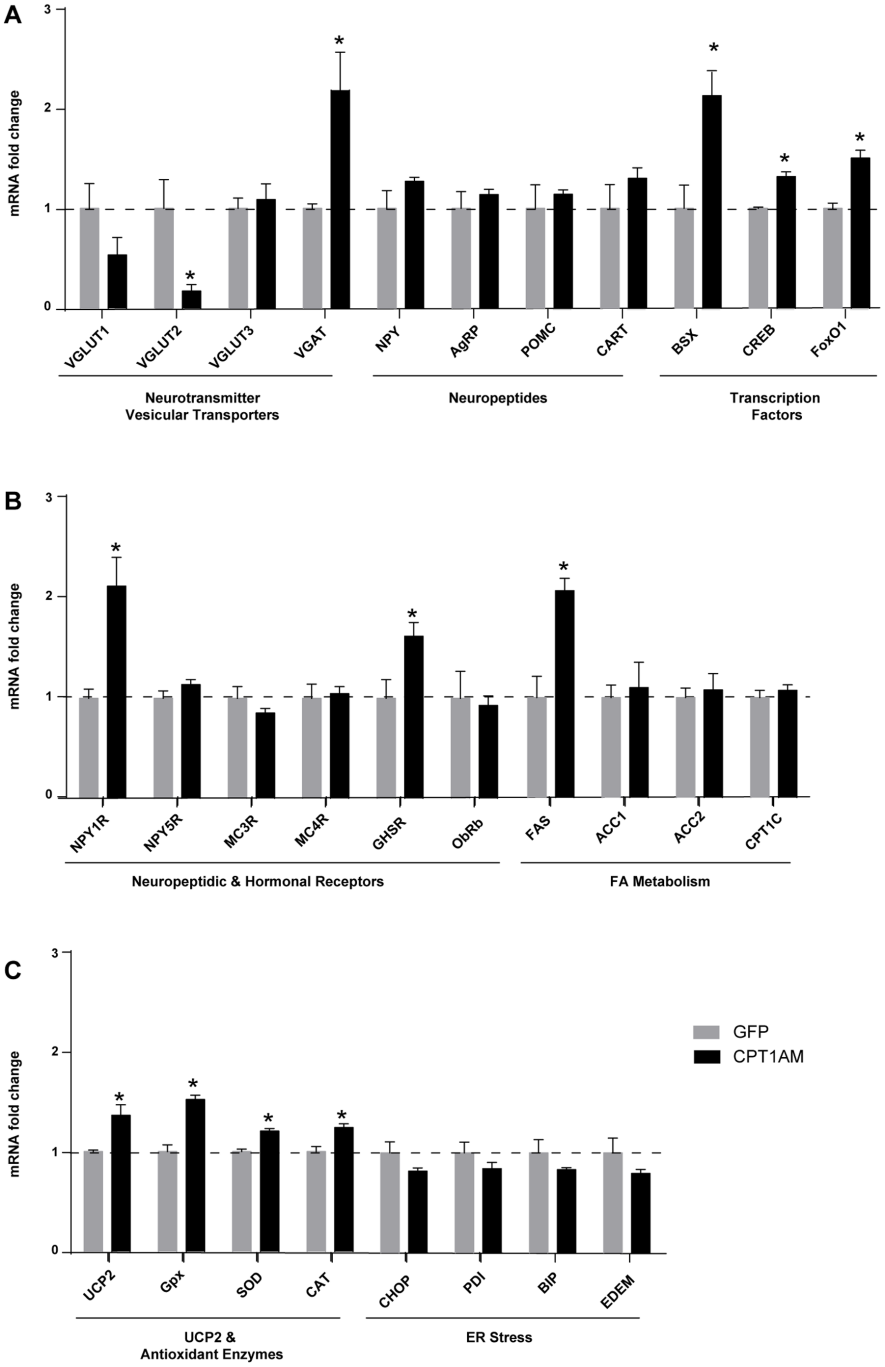
Analysis of MBH gene expression of GFP and CPT1AM rats. All analyses were performed 18 weeks after the AAV-injection into the VMH. (A) MBH relative mRNA expression of hypothalamic vesicular classical neurotransmitter transporters, hypothalamic neuropeptides, transcription factors. (B) Receptors for neuropeptides and hormones involved in feeding regulation and genes associated with fatty acid metabolism. (C) UCP2 and anti-oxidant enzymes and ER stress-related genes. *n* = 6–7 animals per group in all panels. Error bars indicate SEM. *p<0.05.

To discern the molecular mechanisms involved in the hyperphagia produced by CPT1AM expression in the VMH, we first analysed markers of excitatory inputs from the VMH to POMC neurons in Arc in fasted MBH of AAV-infected rats. We observed an 82% decrease (*p*<0.05) in VGLUT2 mRNA levels in CPT1AM rats, without any change in VGLUT1 or VGLUT3. This observation correlates with the finding of unaltered POMC and CART mRNA levels (Fig. 3A) and indicates that the anorexigenic response was not activated.

Vesicular GABA transporter (VGAT) showed a 2.2±0.4-fold increase (*p*<0.05) in the MBH in CPT1AM rats. However, no changes were observed in the mRNA levels of other orexigenic neurotransmitters (such as NPY, AgRP) (Fig. 3A). Interestingly, the mRNA levels of three key transcription factors involved in the expression of the aforementioned orexigenic neuropeptides [21], [37], [38], namely Bsx, CREB and FoxO1, were up-regulated in fasted CPT1AM rats (Fig. 3A) (Bsx: 1.8±0.2-fold increase, *p*<0.05; CREB: 1.3±0.1-fold increase, *p*<0.01 and FoxO1: 1.5±0.2-fold increase, *p*<0.01). Taking into account that the orexigenic neuropeptide NPY exerts its effect on food intake mainly by binding to receptors Y1 (NPY1R) and Y5 (NPY5R), we measured the mRNA levels of these receptors. The NPY1R mRNA level was higher in CPT1AM animals than in controls (2.7±0.5, *p*<0.05) (Fig. 3B), and no changes were observed in NPY5R. Since serum levels of ghrelin were increased in CPT1AM rats, we analysed the mRNA levels of ghrelin receptor (GHS-R), which is known to be induced in fasting conditions [39]. A 1.6±0.2-fold increase in GHS-R (*p*<0.05) was detected, while no changes in mRNA levels of other receptors, such as MC3R, MC4R and ObRb, were observed (Fig. 3B). All these results show that increased VMH CPT1A alters different pathways involved in the control of food intake.

GABAergic transmission is modulated by ROS [40]. Given that CPT1AM expression putatively increases mitochondrial FAO and ROS, we measured the mRNA expression of anti-oxidant enzymes and UCP2 (Fig. 3C). A moderate increase in the mRNA levels of catalase (CAT), glutathione peroxidase 3 (Gpx3), superoxide dismutase (SOD) (1.2±0.04, 1.5±0.1 and 1.2±0.03-fold increase respectively, *p*<0.01) and UCP2 (1.4±0.1-fold increase, *p*<0.05) was detected in CPT1AM animals with respect to GFP controls. However, the markers of ER stress were not induced in VMH CPT1AM rats (Fig. 3C). These data suggest that increased ROS production in the VHM of CPT1AM rats may modulate GABAergic vesicular transporter.

### CPT1AM expression in the VMH alters fatty acid metabolism and the lipidomic profile in the MBH

**Figure 4:**
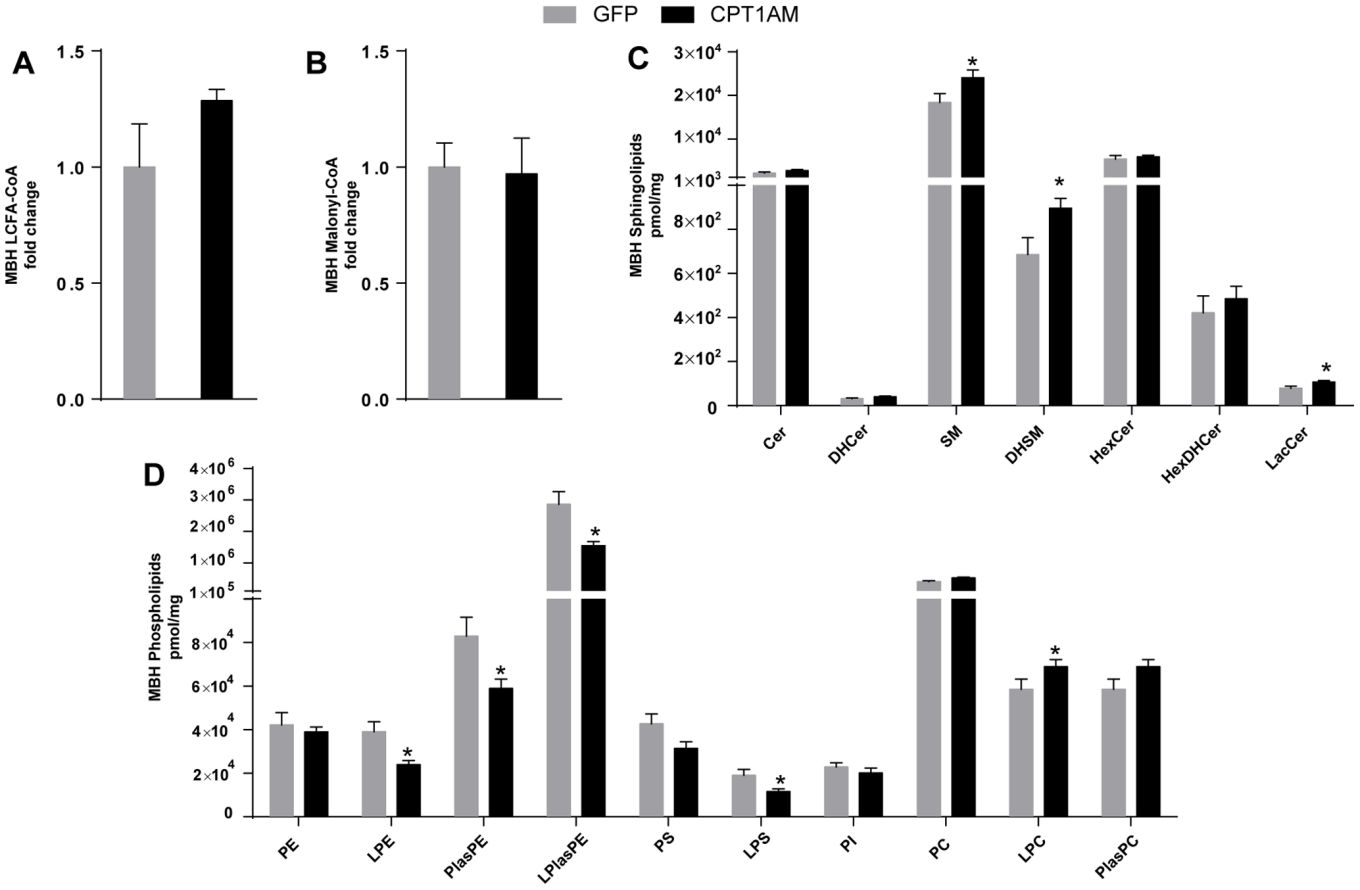
Lipid profile analysis in the MBH of GFP and CPT1AM rats. (A) Relative LCFA-CoA and (B) malonyl-CoA content in the MBH. (C) Sphingolipid (SLs) and (D) phospholipid (PLs) levels in the MBH. *n* = 5-8 animals per group in panels A and B. *n* = 10 animals per group in panels C, D and E. Error bars represent SEM. *p<0.05. DAG: diacylglycerol; TAG: triacylglycerol; Cho-E: cholesterol ester; Cer: ceramide; DHCer: dihydroceramide; SM: sphingomyelin; DHSM: dihydrosphingomyelin; GlucCer: glucosylceramide; GlucDHCer: glucosyldihydroceramide; LacCer: lactosylceramide; PE: phosphatidylethanolamine; LPE: lysophosphatidylethanolamine; PlasPE: plasminogen phosphatidylethanolamine; PS: phosphatidylserine; LPS: lysophosphatidylserine; PI: phosphatidylinositol.

Previous reports indicate that LCFA-CoA levels in the hypothalamus act as a signal in the pathways that modulate food intake. Interestingly, animals with long-term CPT1AM expression in the VMH did not show a reduction of total LCFA-CoA in the MBH (Fig. 4A), in contrast to animals with short-term expression of this isoform [29]. In fact, the levels of C18:0 LCFA-CoA increased in the former (Table 2). We then analysed the mRNA levels of key genes involved in *de novo* FA synthesis (Fig. 4B). CPT1AM animals showed a 2.1±0.1-fold increase in FA synthase (FAS) mRNA (*p*<0.05). Since hypothalamic malonyl-CoA levels have been proposed to regulate feeding, we also measured the concentration of this compound in the MBH of the two experimental groups. Concentrations of this metabolite showed no differences between groups (Fig. 4C). This finding is consistent with the absence of changes in ACC1 and ACC2 between the two groups in the MBH (Fig. 4B). On the basis of these observations, we conclude that the hyperphagic phenotype observed in our model is independent of the variations of these two metabolites.

**Table 2:** LCFA-CoA and ceramides levels in MBH from CPT1AM and GFP rats.

	GFP-expressing rats	CPT1AM-expressing rats	
**LCFA-CoA** (pg/mg protein)	*n* = 5	*n* = 7	*p* value
16:0	1±0.2	1.2±0.1	*ns*
18:0	0.19±0.04	0.3±0.02	0.02
18:1			*ns*
**Ceramides** (pmol/mg protein)	*n* = 10	*n* = 10	*p* value
14:0	5.3±0.7	8.3±1	0.01
14:1	2.7±0.4	4±0.7	*ns*
16:0	66.3±9.6	84.9±13.1	*ns*
16:1	7.3±1.1	9.5±1	*ns*
18:0	1479.9±177.9	1880.8±165.9	*ns*
18:1	170.4±18.8	250.5±31.9	0.02
20:0	246.9±41.3	312.4±23.9	*ns*
22:0	40.3±6.9	51±7.1	*ns*
22:1	10.6±1.3	12.5±1.2	*ns*
24:0	61.2±9.4	67.1±13.5	*ns*
24:1	65±7.8	67.5±10.5	*ns*
24:2	24.3±3.5	29.1±3.6	*ns*

Analysis was performed on MBH extracts obtained at 18 weeks after AAV injection. Data indicated as Mean ± SEM.

To find out whether other bioactive lipids are involved in the orexigenic effect of VMH CPT1AM expression, we performed a lipidomic study of the MBH. Recent evidence has linked ceramide concentration in the hypothalamus to appetite modulation [20], [21]. In our model, total ceramide levels were not significantly different; however, an analysis by species showed an increase in 14:0-ceramide (AAV-GFP, 5.3±0.7 pmol/mg, AAV-CPT1AM, 8.3±1.0 pmol/mg, *p*<0.01) and 18:1-ceramide (AAV-GFP, 170.4±18.8 pmol/mg, AAV-CPT1AM, 250.5±31.9 pmol/mg, *p*<0.02) levels (Fig. 4D, Table 2). We also observed a 36.1% increase in total lactosylceramides (LacCer) in CPT1AM rats (Fig. 4D). Furthermore, other sphingolipids related to ceramides, such as sphingomyelin (SM) and dihydrosphingomyelin (DHSM), were analysed. Total concentrations of SMs and DHSMs were increased by 30.8% and 30.6%, respectively in the MBH of CPT1AM animals.

Membrane traffic and vesicular fusion are especially sensitive to the amount of phosphatidylethanolamine (PE) [41]. We determined the total levels of lysophosphatidylethanolamine (LPE), plasmalogen-phosphatidylethanolamine (PlasPE), and lysoplasmalogen (LPlasPE) and found them to be decreased by 38.5%, 29.0%, 45.8% respectively in CPT1AM rats with respect to controls. The concentration of lysophosphatidylserine (LPS) was also analysed in the MBH, finding it to be decreased by 39% in CPT1A animals (Fig. 4E). Interestingly, the amount of lysophosphatidylcholine (LPC) was 30.0% higher in CPT1AM animals than in controls, even though no changes were observed in total phosphatidylcholine (PC) and plasmalogenphophatidylcholine (PlasPC). Neutral lipids (TAG, DAG and cholesterol esters) were not significantly different between groups (data not shown). All these observations indicate that CPT1A expression in the VMH changes lipid metabolic flows and alters the profile of structural and bioactive complex lipids in the MBH, which may be involved in the hyperphagic phenotype observed.

## Discussion

The hypothalamus is a critical site in the regulation of food intake and energy homeostasis. The VMH has been considered a satiety centre, since lesion of the nucleus induces hyperphagia and obesity [26], [42]. Several years ago, the focus of research into food intake control shifted from the VMH to the Arc with the development of cell-specific molecular tools to study its impact [22], [43], [44]. Nonetheless, the critical role of the VMH in the control of food intake and energy homeostasis has attracted renewed attention. This has arisen as a result of recent studies in knock-out mice in VMH-specific SF1 cells [26], [45], the discovery of neuronal connections between the various hypothalamic nuclei [27], and the crucial role of the Arc- and VMH-originated classical amino acid neutrotransmitters in the control of food intake [46]–[50]. FA metabolism is a key component in the regulation of food intake. We have recently reported that acute expression of CPT1AM, which is insensitive to malonyl-CoA, produces hyperphagia [29]. In the present study we have taken advantage of AAV vectors to demonstrate for the first time that long-term increased CPT1A expression in the VMH produces a chronic hyperphagia and body weight gain. Amongst others, this might be a result of the alteration of vesicular amino acid transporters expression, which are involved in the glutamatergic and GABAergic neurotransmission. Moreover, the alteration on the lipidomic profile found in CPT1AM rats could explain these effects too. These findings emphasise the key role of VMH CPT1A expression in the hypothalamic control of appetite and body weight.

Our data confirm that CPT1AM expression in the VMH produces hyperphagia, as seen by the unsated state of CPT1AM rats. Such chronic over-feeding may contribute to the phenotype observed: CPT1AM rats present overweight and a progression towards insulin resistance, glucose intolerance and hyperglycemia. However, this hyperglycemia may be caused by the direct action of VMH CPT1AM expression. It has been reported that glutamatergic outputs from the VMH exert their effect on liver and thus control gluconeogenesis [51]. This observation is consistent with the observed up-regulation of liver gluconeogenic genes in CPT1AM rats, which show a decreased expression of the glutamatergic VGLUT2. In addition, these animals showed increased adiposity. This centrally driven direct effect may also be involved in the brown-to-white modification of BAT of VMH CPT1AM rats. In support of this notion, VMH has been implicated in the modulation of BAT thermogenesis through parasympathetic innervation [52], [53]. We detected lipid accumulation and hypertrophy in WAT. Although these conditions may be a direct effect of CPT1AM expression in the VMH, we cannot discard indirect mechanisms such as the effect of ghrelin on peripheral tissues [54]–[56].

In our hands, fed VMH CPT1AM rats showed increased circulating levels of the appetite-stimulating hormone ghrelin and NEFA, thus mimicking the metabolic status of fasting animals during the postprandial phase. The relationship between ghrelin hypothalamic signalling and CPT1A activity is well established [22], [23], [29], [56]. Nevertheless, the observation of increased ghrelin levels as a result of VMH CPT1AM expression is striking, since it may indicate a connection between the VMH and the stomach, which is the main producer of octanoylated ghrelin. This notion is reinforced by the observation of reduced ghrelin levels in response to icv administration of C75 [57], a compound that inhibits FAS and CPT1A [35]. Nonetheless, further research is required to confirm these physiological findings.

The molecular mediators in the orexigenic signalling triggered by CPT1A in the VMH are not clear. Although little attention has been given to amino acid neurotransmitters in the control of food intake, their role in hunger signalling has been recently highlighted. Glutamatergic outputs from the VMH have been described to activate anorexigenic POMC/CART neurons to stimulate satiety signalling [27]. In the MBH of rats expressing CPT1AM in the VHM, we observed diminished mRNA levels of VGLUT2, which is the main vesicular glutamate transporter in that nucleus (Fig. 3). Such a decrease may lead to a reduction in glutamate quantal size in the VMH, which would in turn attenuate the activation of anorexigenic neurons. Moreover, optogenetic stimulation on NPY/AgRP neurons produces an orexigenic effect that is blocked using GABA antagonists. This finding indicates that the orexigenic signalling was not dependent on peptidergic neurotransmission [50]. We observed an increase in VGAT expression, which may produce a rise in GABAergic signalling. This inhibitory signalling may come from the VMH [58] and/or from NPY/AgRP neurons [59] to anorexigenic POMC/CART neurons. This notion is consistent with the experiments in which injections of the GABA agonist muscimol markedly increased food intake [60]. Although we do not know how the vesicular transporters are modulated in our model, ROS signalling has been reported to boost GABA release [40]. For this reason, we hypothesise that CPT1AM expression in the VMH, which increases ROS, might be responsible for this higher inhibitory output. Although we did not monitor ROS directly, we observed an increase in the transcription of ROS-buffering enzymes.

**Figure 5:**
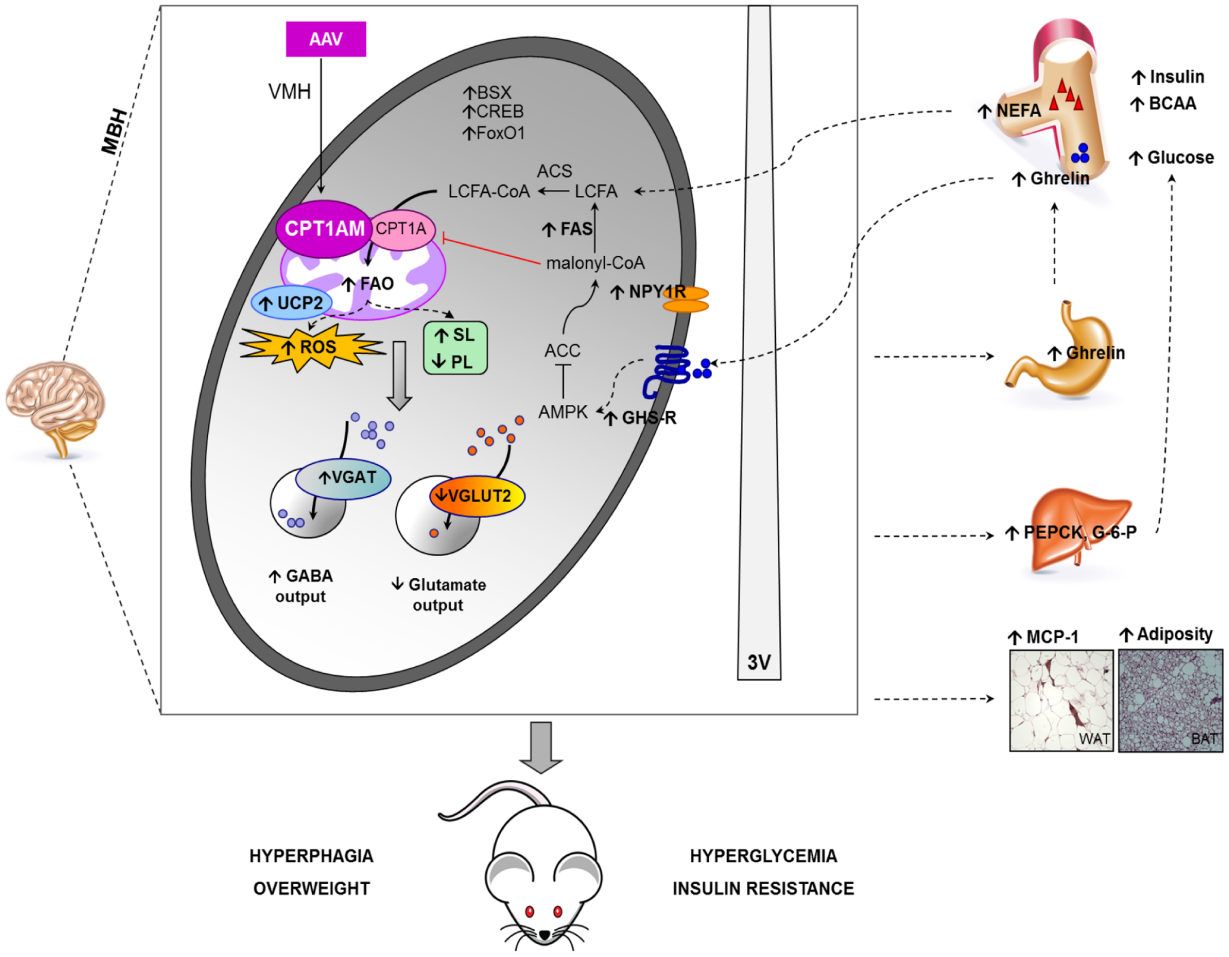
Proposed role of VMH CPT1A in the hypothalamic control of satiety. AAV-mediated CPT1AM expression in the VMH would increase FAO and modulate ROS production and the cellular profile of SLs and PLs. The derived molecular changes in the hypothalamus include the up-regulation of the mitochondrial protein UCP2, the enzyme FAS, and the receptors NPY1R and GHS-R, which indicate an enhanced response to orexigenic NPY and ghrelin. Moreover, an up-regulation of VGAT transporter and a decrease in VGLUT2 may indicate enhanced inhibitory signalling which has been described to promote food intake. The long-term de-regulation of hypothalamic energy sensing induces systemic modifications, including increased circulating levels of BCAA, NEFA, ghrelin, insulin and glucose, the up-regulation of hepatic gluconeogenic genes, increased adiposity, and MCP1 expression in WAT. These central and systemic changes derived from VHM CPT1AM expression promote an increase in food intake and the development of associated metabolic complications.

Alternatively, the lipidomic profile modification driven by CPT1AM expression could be responsible for the increased food intake. CPT1A alters the lipid profile in neurons [61], and membrane composition is crucial to maintain the structure and functionality of embedded proteins [62]–[65]. In some physiopathological states, neurotransmission is modified by alterations in membrane lipid composition [66]. In the synaptic vesicular model developed by Takamori *et al.*, transmembrane proteins encompass one fourth of the whole vesicular surface [64]. Among them, vesicular amino acid transporters, such as VGAT and VGLUT2, require their phospholipidic rims to be anchored to the lipid bilayer and to have a correct functionality [63], [64]. The reduction in phospholipids observed in our model may affect the functionality of these transporters, thus impairing their transcription in some way, at least in the case of VGLUT2. Notwithstanding, we hypothesise that the putative increased VGAT mRNA levels occurs as a result of other factors, such as an indirect effect on other regions within the MBH, mainly Arc, or the aforementioned ROS-induced up-regulation. Both changes in lipid composition and expression of glutamatergic and GABAergic transporters may be implicated in the increase of food intake in animals with CPT1AM expression in the VMH (Fig. 5). Accordingly, we did not observe changes in POMC or CART expression in this animal model or an increase in NPY or AgRP mRNA levels. This could be attributed to the fact that we measured these transcripts in fasting conditions, where both CPT1AM and control GFP rats may express high levels of these orexigenic neuropeptides. However, the transcription factors BSX, CREB and FoxO1, involved in NPY and AgRP expression, were induced in CPT1AM rats [21], [37], [38].

In addition, our results indicate that long-term expression of CPT1AM raises sphingolipid levels in the MBH. It has recently been demonstrated that hypothalamic ceramide metabolism participates in feeding regulation [20]. In particular, the brain-specific CPT1C is required for the fasting-induced increase in hypothalamic ceramides [21]. The present data also implicate CPT1A in ceramide metabolism, as CPT1AM rats showed a significant increase in the hypothalamic concentrations of 18:1 and 14:0-ceramides. Sphingolipids act as signalling molecules in a variety of physiological processes, including neuronal development and plasticity. The formation and transport of specific axonal vesicles has been reported to be coupled to sphingolipid synthesis [67]. Here we observed that CPT1AM expression alters sphingolipid metabolism by increasing SM and DHSM, and it may lead to the modification of hypothalamic synaptic plasticity and energy balance. However, further research is required to test this notion and to clarify the contribution of CPT1A and CPT1C isoforms to hypothalamic sphingolipid metabolism and feeding modulation. In addition, CPT1A expression in the VMH reduces phospholipids in MBH. Although concrete lipid metabolic flows were not the objective of this study, we hypothesise that phospholipids may serve as fuel for increased FAO in CPT1AM-expressing neurons.

Previous hypotheses assume that LCFA-CoAs signal nutrient availability in the hypothalamus and modulate food intake control [14], [15]. Accordingly, in collaboration with Lopaschuck's group [29], we observed that short-term expression of CPT1AM in the VMH produces hyperphagia and reduced LCFA-CoA concentration in MBH neurons. Nonetheless, here we show that total hypothalamic LCFA-CoA content in hyperphagic long-term CPT1AM-expressing animals was similar to GFP control rats and, specifically, the concentration of 18:0-LCFA-CoA was significantly higher in the former. This discrepancy with our previous study might be a consequence of the adaptability of FA metabolism to long-term CPT1AM expression and poses the question as to whether hyperphagia is dependent on LCFA-CoAs levels or whether it relies only on CPT1A activity.

In conclusion, our results indicate that CPT1A expression in the VMH plays a key role in the regulation of food intake and glucose homeostasis (Fig. 5). Mechanistically, our findings suggest that CPT1A modulates mRNA levels of glutamatergic and GABAergic neurotransmission markers and transcription factors controlling orexigenic neuropeptides. Since CPT1AM modifies the composition of sphingolipids and phospholipids and also boosts ROS formation, we cannot discard a mechanistic involvement of these species. Taken together, these data shed light on the central molecular mechanism controlling appetite and highlight mitochondrial FAO in the hypothalamus as a potential target for the treatment of obesity and other eating disorders.
